# Use of impella for off pump myocardial revascularization in high risk patients

**DOI:** 10.1186/1749-8090-10-S1-A160

**Published:** 2015-12-16

**Authors:** Paolo Pepino, Germano Coronella, Salvatore Giordano, Raffaela Provenzano, Agostino La Marca, Mario Monaco, Arturo Giordano

**Affiliations:** 1 Department of Cardiothoracic Surgery, Presidio Ospedaliero Pineta Grande, Castel Volturno, Caserta, Italy; 2 Department of Vascular Surgery, Presidio Ospedaliero Pineta Grande, Castel Volturno, Caserta, Italy; 3 Department of Invasive Cardiology, Presidio Ospedaliero Pineta Grande, Castel Voturno, Caserta, Italy

## Background/Introduction

Patients undergoing coronary surgery are changed in the last years becoming a challenge for the cardiac surgeon not only for the quality of the coronary artery involved in the disease but also for the quality of patients. Low ejection fraction, elderly, COPD, kidney failure, Diabetes are often found in patients with coronary artery disease undergoing CABG.

## Aims/Objectives

Beating heart myocardial revascularization is a well-established surgical technique but in case of very low EF (below 30%) there is a particular high surgical risk of conversion to on-pump surgery. The recently developed of Impella device (Abiomed, Danvers, MA) represent a compromise between circulatory support and limited invasiveness.

## Method

Three patients, male, admitted for acute coronary syndrome with three vessels CAD and low ejection fraction below 30 %, were operated in our department. After preliminary lower limb duplex ultrasound to exclude peripheral artery disease, an Impella Recover LP 5.0 device was inserted via surgical exposure of the right femoral artery in one case and an Impella recover LP 4,5 via percutaneous insertion of the femoral artery in two cases.

## Results

The myocardial revascularization was performed using a complete arterial revascularization: Left internal mammary artery on the LAD and the radial artery "T" anastomosed from the LIMA to the other vessels (diagonal, intermedius ramus, OM and PDA). The device was left in for 48 Hours after the operation. All patients were discharged home after 9-10 days after admission and a follow up echocardiography performed six month after the operation showed an increase of the ejection fraction up to 40-45%.

## Discussion/Conclusion

Use of the Impella Recover device for patients undergoing off pump CABG is feasible and safe, and appears a promising strategy to improve short- and long-term outcomes.

